# Multiple Talon Cusps and Dens Evaginatus in the Maxillary Dentition With Coexisting Single-Tooth Hypodontia: A Case Report

**DOI:** 10.7759/cureus.102727

**Published:** 2026-01-31

**Authors:** Kostis Giannakopoulos, Anna Digka, Michalis Kathopoulis, Cima Sater, Efthymios Karayiannis

**Affiliations:** 1 School of Dentistry, European University Cyprus, Nicosia, CYP

**Keywords:** dens evaginatus, dental anomalies, dental morphology, hypodontia, talon cusp, tooth morphology, tubercle

## Abstract

This article presents a rare case of a young adult with multiple talon cusps on maxillary anterior teeth, dens evaginatus on maxillary second premolars, and hypodontia of the mandibular second premolar. These anomalies can cause occlusal interference, caries, fractures leading to pulp exposure, and esthetic concerns. Management options include gradual cusp reduction, composite resin coverage, pulpotomy, apexification, root canal therapy, or no treatment when asymptomatic.

A 20-year-old male patient presented with pain in the left mandibular wisdom tooth. Examination revealed talon cusps on all six maxillary anterior teeth and dens evaginatus on both maxillary second premolars. The mandibular second premolar was congenitally absent, with retention of the primary molar. As there were no complications related to the anomalies, regular monitoring was advised. This case highlights the importance of thorough examination, early detection, and individualized management of dental anomalies to prevent complications.

## Introduction

A talon cusp (TC) is an uncommon developmental alteration of tooth morphology, in which an additional cusp-like structure arises from the cingulum or the cemento-enamel junction (CEJ) and extends toward the labial or lingual surface of anterior teeth in either jaw [1]. The same anomaly observed on posterior teeth, typically premolars, where a supernumerary tubercle protrudes from the occlusal surface, is referred to as dens evaginatus [2]. Although both entities are considered rare in the general population, their clinical relevance may complicate diagnosis and treatment planning.

From a clinical perspective, these accessory cusps may contain enamel, dentin, and in some cases pulpal tissue, increasing the risk of occlusal interference, premature contacts, attrition or fracture, caries development in deep developmental grooves, and pulpal complications. They may also interfere with orthodontic alignment, space management, and restorative procedures, particularly when multiple teeth are involved or when the anomalies coexist with other developmental disturbances. For this reason, early recognition and individualized management are important to prevent avoidable complications and to preserve tooth vitality whenever possible [3].

This article aims to report a case of a young adult with multiple TCs on maxillary anterior teeth, dens evaginatus on second maxillary premolars, and hypodontia of tooth #35. The simultaneous occurrence of multiple anterior TCs with posterior dens evaginatus and single-tooth hypodontia is unusual and noteworthy, as it represents a rare combination of odontogenic anomalies within the same patient and may pose specific diagnostic and management challenges.

## Case presentation

A 20-year-old Caucasian male patient presented to the European University Cyprus dental clinic complaining of pain around tooth #38 and noticeable calculus accumulation. His medical history included treated chronic asthma, for which he received corticosteroid treatment until the age of 13. He reported no other medical conditions, was not taking any medication, and had no known allergies. The patient's lifestyle habits included daily smoking, bi-weekly consumption of sweets, weekly alcohol intake, and daily consumption of carbonated drinks. Oral hygiene practices included brushing once daily and flossing every two days.

**Figure 1:**
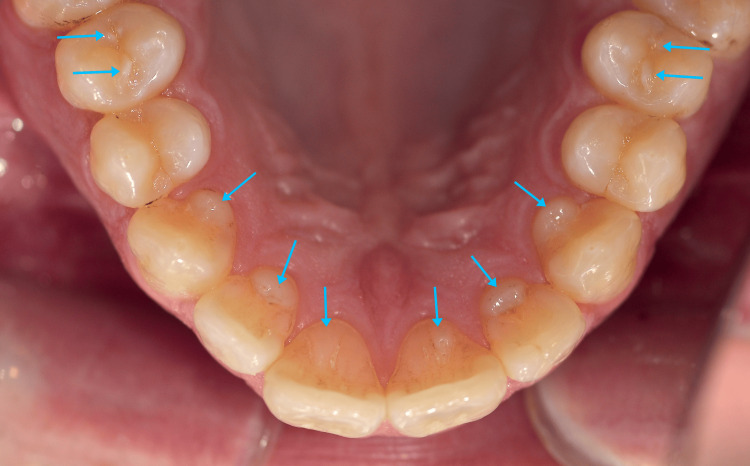
Talon cusps on all six maxillary anterior teeth (Type II on teeth #13, #12, #22, and #23; Type III on teeth #11 and #21). Note the dens evaginatus on the maxillary second premolars (teeth #15 and #25).

**Figure 2:**
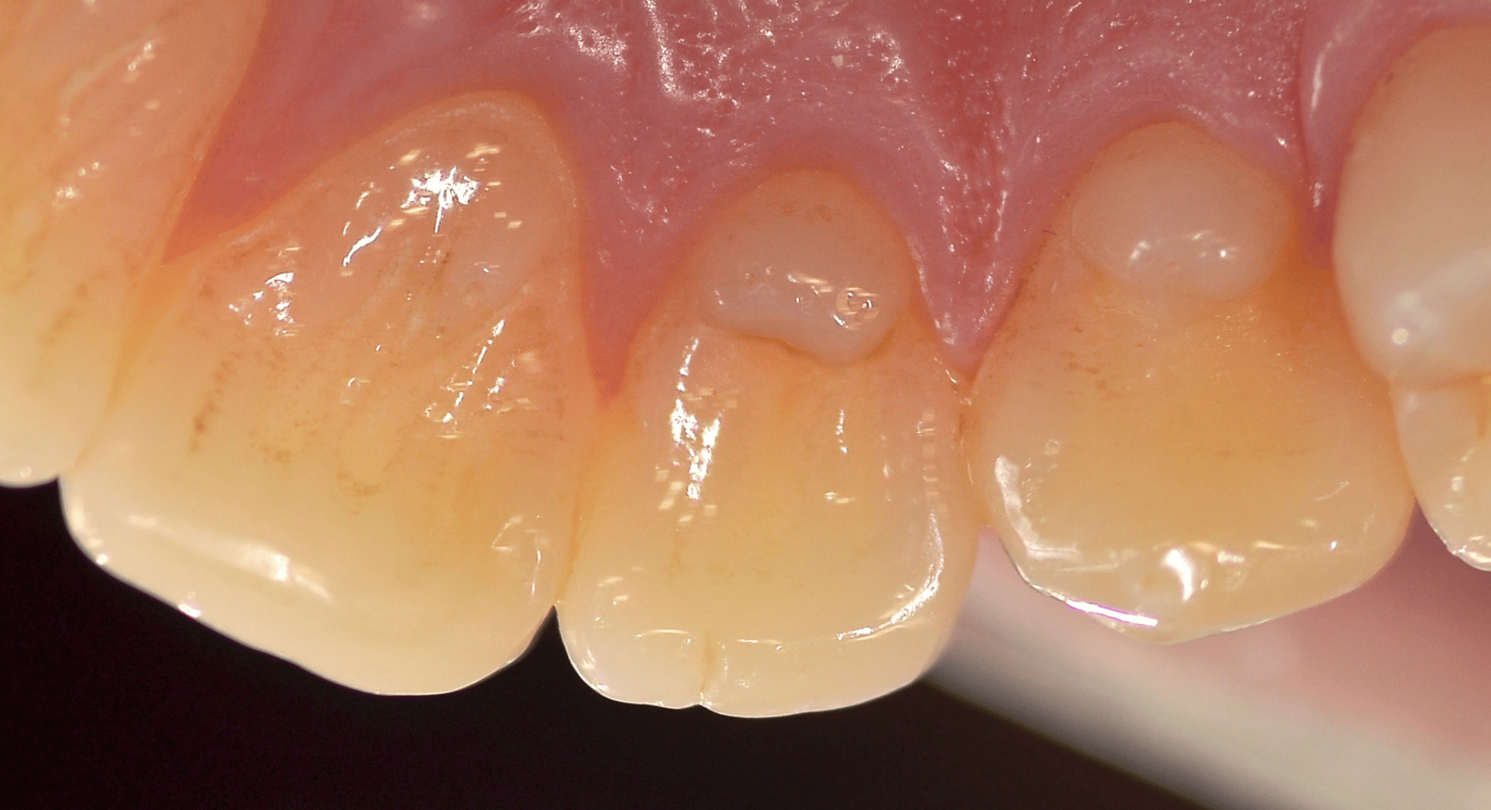
Close-up photograph of the talon cusps on teeth #21, #22, and #23. Note that the talon cusp on the central incisor is significantly long inciso-cervically and thinner facio-lingually.

Clinical examination revealed slight gingival inflammation around mandibular anteriors and distal to tooth #37. Teeth #46 and #47 exhibited occlusal caries of International Caries Detection and Assessment System (ICDAS) code 2. Plaque accumulation was observed on mandibular incisors, along with staining from smoking on lingual surfaces of maxillary anteriors. Composite resin restorations were present on teeth #16, #26, and #36. Enamel projections were prominent on the lingual surfaces of teeth #13, #12, #11, #21, #22, and #23, consistent with TCs (Figures 1, 2).

**Figure 3:**
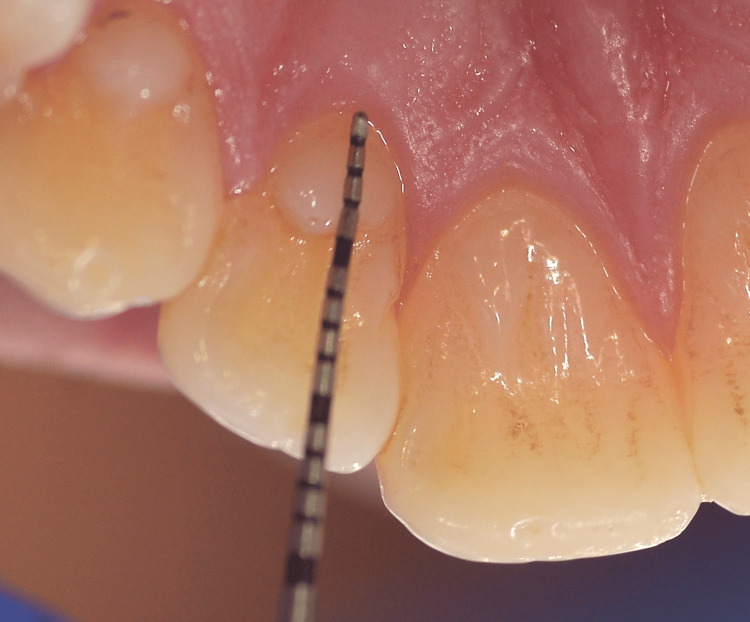
Measurement of talon cusp dimensions using a North Carolina periodontal probe, with the probe held parallel to the long axis of the tooth to record inciso-cervical length. The mesio-distal and facio-lingual dimensions were also measured with the same probe.

TC dimensions were measured using a North Carolina periodontal probe, with the probe held parallel to the long axis of the tooth; all measurements were taken twice by the same examiner, and the mean values were recorded (Figure 3). After measuring the dimensions of the TCs, they were classified according to Hattab et al. (Table 1) [3].

**Table 1:** The dimensions of the talon cusps (TCs) and the classification of the anomalous structures according to Hattab et al. [3]. Crown length was measured inciso-cervically from the incisal edge to the CEJ on the palatal surface. CEJ: cemento-enamel junction

Tooth #	TC length	TC mesio-distal width	TC facio-lingual dimension	Crown length	Classification
13	3 mm	3 mm	3 mm	9 mm	Type II
12	3 mm	4 mm	2 mm	10 mm	Type II
11	4 mm	2 mm	1 mm	11 mm	Type III
21	4 mm	2 mm	1 mm	11 mm	Type III
22	3 mm	4 mm	2 mm	10 mm	Type II
23	3.5 mm	4 mm	3 mm	9 mm	Type II

**Figure 4:**
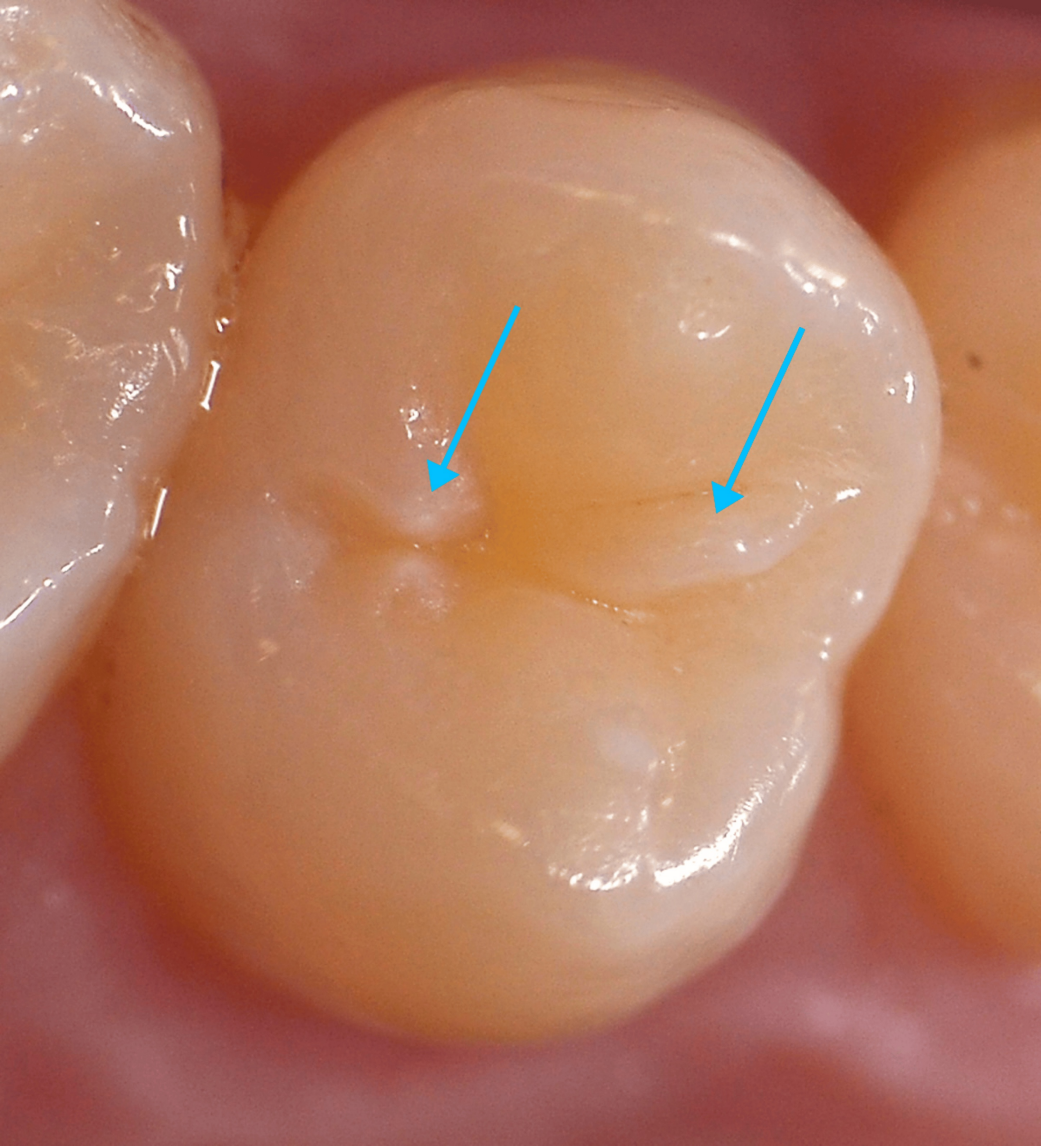
Close-up photograph of tooth #25. A larger enamel projection is attached to the mesial cusp slope of the buccal cusp, and a smaller one is attached to the distal cusp slope of the buccal cusp, interrupting the transverse ridge.

Further to that, enamel projections were apparent on each of the second maxillary premolars (teeth #15 and #25), a larger one attached to the mesial cusp slope of the buccal cusp, and a smaller one attached to the distal cusp slope of the buccal cusp, interrupting the transverse ridge (Figure 4).

**Figure 5:**
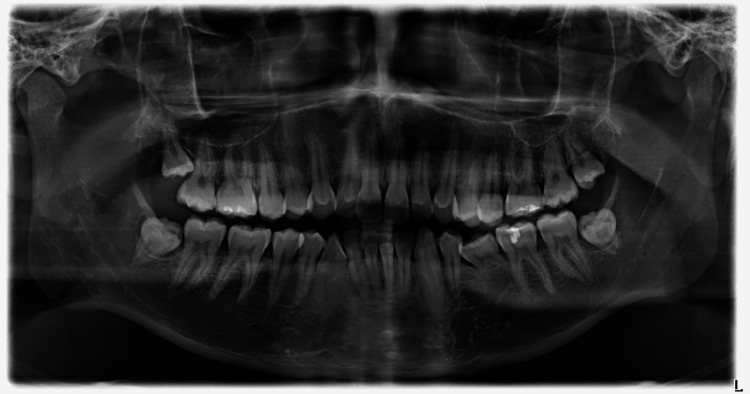
Panoramic x-ray of the patient, where the six maxillary anterior talon cusps appear clearly. The retained primary left second molar, tooth #75, appears to have partially resorbed roots, due to the congenitally missing tooth #35. As there are no clinical signs of mobility, the tooth may be retained for an unknown time in the mouth.

Tooth #75 was retained and appeared ankylosed. Soft tissues were found to be within normal limits. Radiographic assessment with a panoramic radiograph confirmed the presence of TCs on all six maxillary anterior teeth, extending toward the incisal edge (Figure 5).

Additionally, the mandibular left second premolar, tooth #35, was congenitally absent. No periapical radiolucencies or other radiographic signs suggestive of pulpal or periradicular pathology associated with the accessory cusps were observed. The retained primary mandibular left second molar (#75) showed partial root resorption radiographically, without clinical signs of mobility or symptoms at the time of examination.

For this patient, no treatment was provided for the TCs and dens evaginatus as there were no symptoms or esthetic concerns reported, no occlusal interference on clinical examination, and no radiographic signs suggestive of periapical pathology. The patient was fully informed and scheduled for follow-up at six-month intervals, and when indicated, other treatment options should be considered.

## Discussion

The term "talon cusp" was introduced in 1970 by Mellor and Ripa, who likened its appearance to that of an eagle's talon in axial view [4]. One of the most commonly used classification systems was recommended by Hattab et al. in 1996, which categorizes the anomaly into three types [3]. In Type I (talon), a well-defined accessory cusp arises from the lingual surface of an anterior tooth in either the primary or permanent dentition and reaches approximately one half or more of the crown height, measured from the CEJ toward the incisal edge; Type II (semi-talon) describes a smaller cusp-like projection, typically at least 1 mm in size, that extends less than half of the clinical crown and may appear either partially integrated with the palatal surface or clearly demarcated from the remainder of the crown. Type III (trace talon or prominent cingulum) encompasses subtle morphologic variations, presenting as an enlarged or irregular cingulum, including conical, bifid, or tubercle-like forms.

The prevalence of TC anomaly varies in the literature from less than 1% to almost 8%, and it appears to be geographically dependent and may be more common in people of Asian origin [1,5,6]. Radiographically, the overall prevalence of TCs was 0.23% of all the teeth evaluated in a radiographic survey of 15,000 teeth [7]. TC most commonly appears in maxillary anterior teeth and specifically lateral incisors (55%) and central incisors (33%), and less frequently on mandibular canines (6%) and maxillary canines (4%) [8]. Consistent with previous reports, TCs affect the maxillary anterior teeth, whereas dens evaginatus is typically described in premolars [2,8]. In the present case, the TCs were classified as Type II and Type III according to Hattab et al., reflecting the variability in morphology and extent reported in the literature [3].

The etiology of the TC is believed to involve both genetic and environmental components. From a developmental perspective, the anomaly has been associated with localized disturbances in odontogenesis, potentially arising from abnormal folding of the inner enamel epithelium during crown formation or from temporary, focal proliferation of mesenchymal tissue [3,9]. A genetic contribution has further been proposed to account for the frequent concurrence of TC with other dental developmental anomalies [10].

Although TCs commonly occur as isolated findings, they are often associated or coexist with other dental developmental abnormalities such as fused teeth, congenitally missing or supernumerary teeth, ectopic or impacted canines, inverted or erupted mesiodens, radicular anomalies, complex odontomes, dens evaginatus of posterior teeth, peg-shaped and shovel-shaped lateral incisors, and exaggerated Carabelli’s cusps [11-13]. Also, it appears in the oral manifestations of Rubinstein-Taybi syndrome, Temtamy preaxial brachydactyly syndrome, hypomelanosis of Ito syndrome, and Mohr syndrome (orofacial digital syndrome, Type II) [14].

Several authors suggest that clinical problems noted with TC cases include attrition, compromised esthetics, occlusal interferences, temporomandibular joint pain, accidental cusp fracture, soft tissue discomfort involving the tongue during functional activities such as speech, mastication, periodontal complications related to excessive occlusal loading on the talon, and an increased susceptibility to caries within the developmental groove associated with the accessory cusp. In some cases, extension of the anomaly toward the root surface has been reported, potentially contributing to localized periodontal inflammation. Moreover, pronounced TCs may interfere with normal mandibular development, predisposing affected individuals to a Class II malocclusion [15].

On radiographic examination, particularly in cases involving unerupted teeth or odontoma-like presentations, TCs may be mistakenly interpreted as supernumerary teeth, potentially resulting in unwarranted surgical intervention [16]. Therefore, careful differential diagnosis is required, with consideration given to conditions such as dens invaginatus and mesiodens [17].

Treatment modalities for TCs are employed on a case-by-case basis, depending on clinical implications, patient symptoms, and preferences. Possible treatment plans include intermittent grinding of the TC or dens evaginatus before any fracture and pulpal involvement occurs [2], covering the TC with composite resin to reinforce and protect it, coronal pulpotomy, endodontic management through apexification followed by obturation, conventional root canal treatment in fully developed teeth, surgical endodontic approaches, or tooth extraction when indicated as part of an orthodontic treatment plan. In selected cases, active intervention may be unnecessary, particularly when neither occlusal interference nor a significant risk of cusp fracture is present [2,15,18-20].

TCs and dens evaginatus are developmental dental anomalies that rarely coexist in the same patient, and we could retrieve only one reference with both anomalies present [3]. Furthermore, the presence of congenitally missing teeth adds another layer of rarity to such cases. In this article, we present a unique case of a young adult male patient with multiple TCs on maxillary anterior teeth, dens evaginatus on second maxillary premolars, and hypodontia of tooth #35.

This case highlights the importance of thorough clinical and radiographic examinations in identifying and managing dental anomalies so that appropriate therapeutic or preventive clinical decisions can be made, based on proper diagnosis. Different approaches to treatment have been reported, customized for the specific patients and, in general, with good results [21]. Selectively grinding the TC or dens evaginatus in 6-8-week intervals, followed by fluoride varnish application, was successful, without any sensitivity or other adverse effects taking place [15]. Talon preservation or coverage with composite has also been reported to be successful [15].

## Conclusions

A rare case of a 20-year-old male patient with multiple TCs on maxillary anterior teeth, dens evaginatus on second maxillary premolars, and hypodontia of tooth #35 is presented in this paper. This case represents a combination of developmental dental anomalies, which is seldom reported in the literature.

Regular follow-up and preventive measures are crucial in the management of such cases to prevent complications and optimize oral health outcomes. Through comprehensive history-taking, clinical and radiographic evaluation, and interdisciplinary collaboration, dental practitioners can effectively manage patients with complex dental anomalies. In asymptomatic cases, a conservative approach with periodic review (e.g., six-month intervals) is appropriate, with intervention indicated if occlusal interference, caries in the developmental grooves, cusp fracture/wear, pain or sensitivity, or radiographic signs of pulpal/periapical pathology develop.
